# Somatotypes and hand-grip strength analysis of elite cadet sambo athletes

**DOI:** 10.1097/MD.0000000000018819

**Published:** 2020-01-17

**Authors:** Tatjana Trivic, Sergey Eliseev, Sergey Tabakov, Vuk Raonic, Cristina Casals, Dzenan Jahic, Damjan Jaksic, Patrik Drid

**Affiliations:** a Faculty of Sport and Physical Education, University of Novi Sad, Serbia; b Russian State University of Physical Education, Sports and Tourism, Moscow, Russia; c MOVE-IT Research Group and Department of Physical Education, Faculty of Education Sciences; d Biomedical Research and Innovation Institute of Cadiz (INiBICA) Research Unit, Puerta del Mar University Hospital, University of Cadiz, Cadiz, Spain; e Orthopaedics and Traumatology Clinic, University Clinical Center Sarajevo, Sarajevo, Bosnia and Herzegovina; f Faculty of Sport and Physical Education, University of Montenegro, Podgorica, Montenegro.

**Keywords:** body weight, hand strength, martial arts, skinfold, somatotypes

## Abstract

The objectives of this research were to establish somatotype and hand-grip strength between elite cadet male and female sambo athletes divided by weight categories.

A total of 97 elite cadet sambo athletes, participants of the World Cadets Sambo Championships 2018 participated in the study. Male and female sambo athletes were divided by official weight categories. Anthropometrical variables were taken in order to calculate somatotypes and hand-grip strength. A one-way analysis of variance and Tukey's post hoc tests were used to compare group differences by weight categories.

Results of this study provide the first description of somatotype and hand-grip strength of elite male and female cadet sambo athletes in relation to weight category. A typical somatotype in male sambo athletes was endomorphic mesomorphs with a predominance of musculoskeletal tissue, while female athletes differed concerning weight category. Overall, an increase in handgrip strength across weight categories was noted. Hand-grip strength increases linearly from the lightest to the heaviest weight category except in −66 and −84 kg in male athletes. Differences in handgrip strength of female athletes were detected between the lightest group and last six groups in all three variables in favor of last six as well as −44 and kg −48 kg compared with the heaviest.

To the best of our knowledge, this study provides the first normative data of somatotype and hand-grip strength analyses in relation to age, gender, and weight categories of cadet sambo athletes. The anthropometric profile of sambo athletes changed according to their weight category. Mesomorphy was the most dominant somatotype component in male athletes, while female had three different types of somatotype component in relation to weight category. In conclusion, we found differences in hand-grip strength related to weight category, which can be linked to the muscle mass of athletes. Future studies should focus on somatotype and strength handgrip values of international compared to national level sambo athletes.

## 1 Introduction

In 1938 the Committee of Sports of Union of Soviet Socialist Republics accepted Sambo as the official combat sport of the Soviet Union. After 80 years of existence and development sambo has received temporary recognition from the International Olympic Committee (IOC), and makes its first step towards inclusion at the Olympic Games. Sambo is a sport based on the specific age and weight categories. Weight categories are an important factor in determining the morphological differentiation of sambo competitors. There are 10 weight categories for cadets in sambo competition for each gender. According to the category-specific demands, differences in technique, tactics, physiology, and functional aspects have been studied in similar combat sports.^[1]^ There is only one study in which body structure and somatotypes of elite sambo athletes have been evaluated.^[2]^ The training process of qualified sambists (sambo athlete) is quite lengthy and depends on a large number of different factors.^[3]^ Anthropometric characteristics and maximal strength represent essential elements of physical performance in sambo. Also, one of the goals in sambo training is to increase muscle strength, maximize lean tissue and minimize body fat. However, to the best of our knowledge, there is no available information in the literature about age-related somatotype or hand-grip strength in cadet-athletes. In weight classified sports, athletes usually try to minimize adiposity in early adolescence.^[4]^ However, research related to young athletes in sambo are still in the development phase. Recent studies are predominantly focused on elite athletes who have already reached a high standard of performance, with a need for further improving and developing the methodological basis of sambo training.^[5]^

Grip strength is an important component of many combat sports.^[6–9]^ In sambo, good grip control enables athlete to execute a throwing technique or give the opportunity to continue the action on the ground position. Success in sambo requires a high level of physical and tactical preparation, regardless of the weight category. In combat sports, strength is known to increase competition success, especially, handgrip strength (grasping strength) is a very important determinant.^[10]^ During the sambo match, most of the time is spent on gripping the opponent's jacket (sambo uniform), and fighting for adequate grip usually results in high levels of fatigue in the forearms.^[11]^ Previous studies in combat sports show that grip strength is positively correlated to stature, somatotype components, and anthropometric measurements.^[12]^ Also, combat sport studies have observed lower body fat percentages and greater strength in elite compared to sub-elite combat athletes.^[13,14]^ Although sambo is a sport with a long history and is recognized from the IOC, it is necessary to define minimum fitness standards required for success in all age categories. Therefore, we investigated the relationship between somatotype and hand-grip strength in highly trained cadet sambo athletes of both genders, divided by weight category. This research aims to provide first-ever somatotype models associated with handgrip strength of elite cadet sambo athletes by each weight category.

## 2 Methods

### 2.1 Study design

This was a cross-sectional and exploratory study.

### 2.2 Sample size calculation

A total of 203 elite cadet sambo athletes from 28 countries participated in the World Cadet Sambo Championships 2018 in Novi Sad, Serbia. For the purpose of this study, we have chosen 97 sambo athletes. Of the total number of registered competitors, 47.8% was recruited in this study. Out of a total number of female sambo athletes in the competition, 57.74% participated in the study and out of the total number of male sambo athletes 42.42% took part in the study. According to the calculation, considering that the 10 weight categories are analyzed, the total sample size should be 390. However, in this specific case, the total population is 203 competitors, so the classical formula cannot be applied. In that meaning, non-parametric statistics are used in addition to parametric statistics.

### 2.3 Participants

This study consisted of 41 female sambo athletes (age 15.64 ± 0.79 years) and 56 male sambo athletes (age 15.94 ± 0.83 years). According to International Sambo Federation (FIAS) only athletes aged 15 to 16 years were allowed to compete in World Championship for cadets. Measurements were taken in November 2018. All testing procedures were conducted during the World Cadet Sambo Championship held in Novi Sad (Serbia). Participants were divided into ten official male (−42, −46, −50, −55, −60, −66, −72, −78, −84, and +84 kg) and female categories (−38, −41, −44, −48, −52, −56, −60, −65, −70, and +70 kg). All participants were divided into weight categories in accordance with their age and sex in accordance with the FIAS International sambo competition rules. All participants took part in the study on a voluntary basis. Participants were familiarized with all testing procedures used in the present study. Four graduate students of Faculty of Sport and Physical Education with experience have measured the same measure in all 3 days of competition at the same position. Informed written consent was obtained from each subject, and all procedures were performed in accordance with the Declaration of Helsinki. The study was approved by the local Institutional review board of Faculty of Sport and Physical Education, University of Novi Sad (Ref. No. 548/2018; approved at October 1, 2018).

### 2.4 Anthropometrical measurements

Following anthropometric measurements were conducted: height and body mass, four skinfolds (triceps, subscapular, supraspinale, calf), breadths (humerus and femur diameters), girths (arm and calf), breadths (humerus and femur diameters). Body height was determined using a Martin anthropometer (GPM, Switzerland), skinfolds were measured using a John Bull caliper (British Indicator Ltd, UK) accurate to 0.2 mm, girth measurements were acquired with a steel measuring tape, and wrist girth and bicondylar diameters of the femur and humerus were measured using a small spreading caliper (SiberHegner, Switzerland). Somatotypes were determined according to the Carter and Heath method (1990).^[15]^

### 2.5 Hand-grip strength

Maximum handgrip strength for both hands was measured with a portable Takei handgrip dynamometer (Takei Scientific Instrument CO., Tokyo, Japan).

### 2.6 Statistical analysis

Data are presented as means and standard deviation (±). A one-way analysis of variance and Tukey's post hoc tests were used to compare group differences by weight categories. In cases where Kolomogorov–Smirnov test shows statistically significant differences from normal curve, deference between occurred weight category and any other categories was calculated with Mann–Whitney *U* test. Effect size (η^2^) was calculated as well. The level of significance was set at 5%. All analyses were conducted using SPSS statistics software.

## 3 Results

All sambo athletes, who were recruited for the present study, participated in World Cadet Sambo Championships held in Novi Sad and modified their body mass regularly to compete in the adequate weight category. In all male sambo categories, there were a minimum number of two participants, which gave us the possibility for comparing all group pairs (Table 1).

**Table 1:**
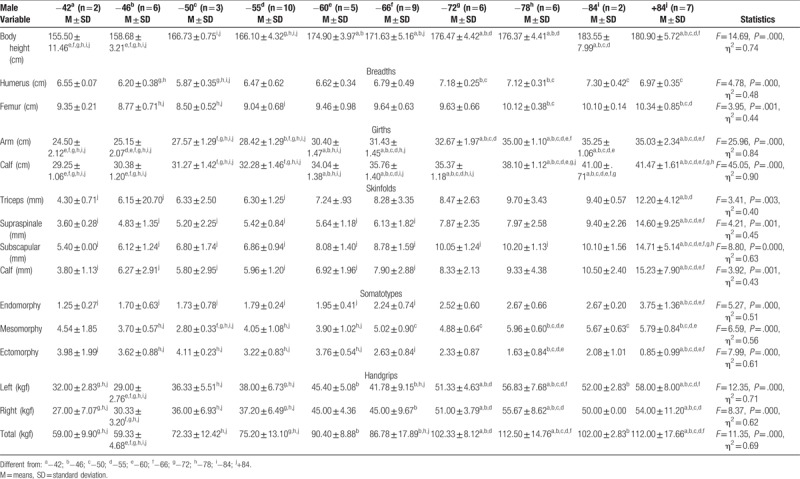
Differences in anthropometric variables and handgrip strength between weight categories of elite cadet male sambo athletes.

**Figure 1:**
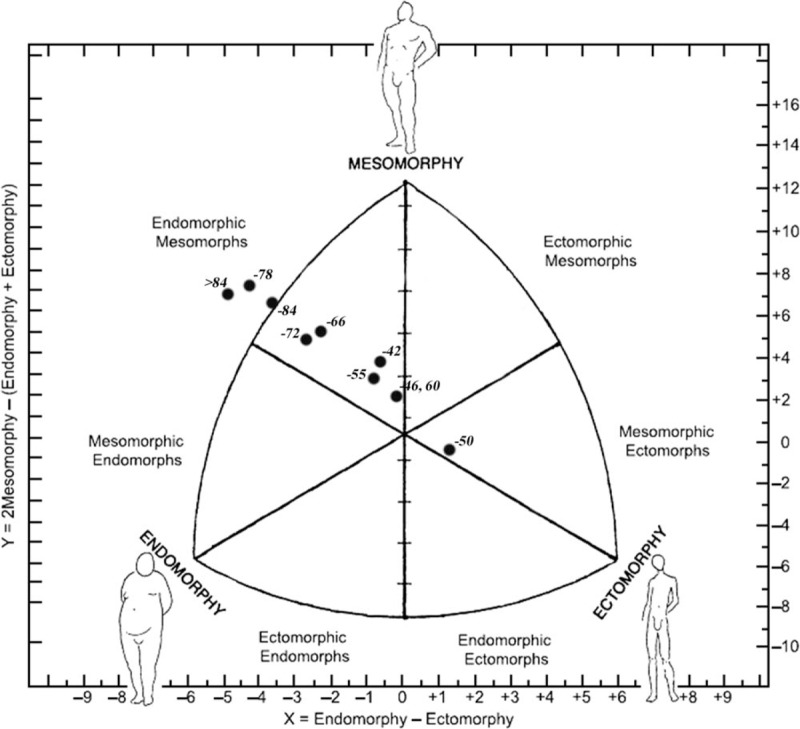
Somatochart of cadet elite male sambo athletes by weight categories.

The first two weight categories showed significant differences in height compared to last six weight categories. Also, body height increased in proportion with weight categories. Statistically significant differences in humerus breadth were found between −46 kg, −50 kg and −72 kg, −78 kg and as well as between −84 kg, above 84 and −50 kg. Femur breadth showed differences between the heaviest group of athletes and −46, −50, and −55 kg and also between −78 and −46 kg and −50 kg. In the domain of girths, it could be concluded that almost all group pairs are statistically different from each other. In terms of skinfolds, all male weight categories, except −84 kg showed differences predominately with the heaviest category. Somatotype analysis showed noticeable differences between the heaviest weight category and first six weight categories of sambists in endomorphy and ectomorphy component. Differences in mesomorphy component are also noticeable between the second to last weight categories, where the main differences are observed in −78 kg and above 84 kg (Fig. 1). There was a significant effect of weight category for the right, left and total hand-grip strength (Table 1). An effect of weight category for the left handgrip strength was noted with the lower values for the −46 kg compared to last six categories, while higher values were observed in −78 kg and above 84 kg in compared with first five categories. An effect of weight category for the right handgrip strength was also noted but generally obtained values were lower in comparison with left handgrip strength, except in the −46 and −66 kg. In sum, a similar difference was found as for a left hand.

In female athletes, body height from first four and sixth weight category was different compared with the heaviest weight category as well as between −38 and −70 kg (Table 2).

**Table 2:**
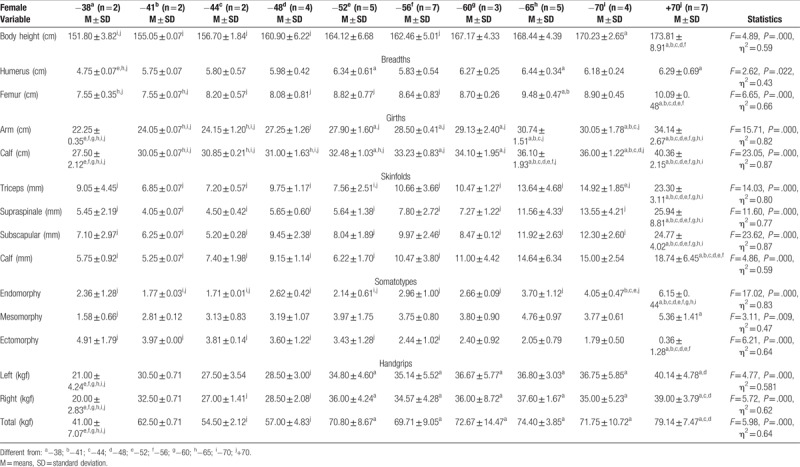
Differences in anthropometric variables and handgrip strength between weight categories of elite cadet female sambo athletes.

**Figure 2:**
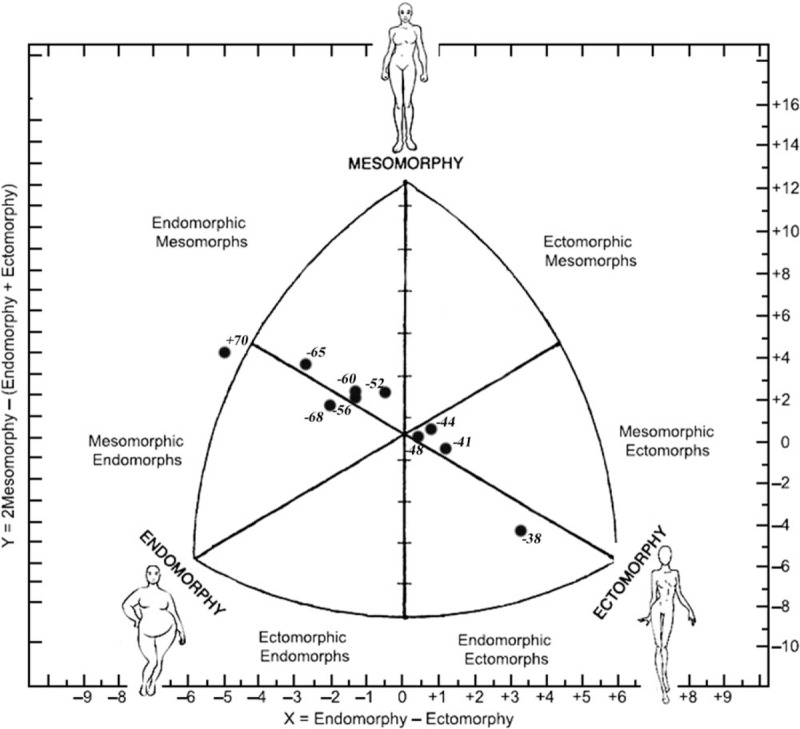
Somatochart of cadet elite female sambo athletes by weight categories.

Humerus breadth was different between the lightest weight category, −52 kg, −65 kg, and above 70 kg, but femur breadth showed differences between the heaviest and first 6 groups. In terms of skinfolds, differences were found between the heaviest group and all the others, as well as for both girths. Except that in domain of girths, it could be seen that the lightest group was significantly different when compared to almost all other groups. Somatotype analysis has found differences in mesomorphy component between the lightest and the heaviest group. Endomorphic component of the heaviest weight category was different comparable to all others as well as in terms of ectomorphy where the differences were found between the heaviest and first six weight category (Fig. 2). In domain of hand grip, strength differences between weight categories in female athletes were found between the lightest group and last six groups in all three variables in favor of last six as well as for −44 and −48 kg compared with the heaviest weight category (Table 2).

## 4 Discussion

The comparison of specific characteristics of athletes allows estimating the influence specificity of sport and identifying criteria for success in sport. According to our knowledge, this is the first research that investigates anthropometric characteristics associated with handgrip strength of elite cadet competitors of both genders. Due to the specific requirements of sambo, obtained results of this study will provide useful information that relates to each weight category. Several physical characteristics were noted in sambo athletes between different weight categories and gender. Considering the basic anthropometrical characteristics, higher average height is observed with the increase in weight of both genders. Analysis of bone diameters demonstrated a linear increase from the light to the heavy weight categories. Exception in linear increase for humerus breadth was found in male −50 kg, −78 kg, and above 78 kg weight category. In female athletes humerus breadth was different between the lightest weight category, −52 kg,−65 kg, and above 70 kg. Statistically significant differences in femur breadth showed differences between the heaviest group of athletes and −46, −50, and −55 kg and also between −78 kg and −46 kg and −50 kg. Contrary, femur breadth in female athletes showed differences mainly between the heaviest and first 6 groups. Concerning girths, it could be concluded that almost all weight categories are statistically different between each other. Male sambo athletes demonstrated a linear increase from the light to the heavy weight category. This trend is also evident among female athletes. Optimal body composition is a relevant aspect in sambo since competitors are divided into weight categories. Also, higher levels of body fat are negatively correlated with the performance of locomotion.^[16–18]^ In this regard, control of body composition is necessary to define athlete's best weight category. However, process of weight loss starts in early adolescence in both genders, and has long been a matter of concern.^[4,19]^ According to somatotypes, male sambo athletes showed mostly endomorphic mesomorph somatotype, excepts for −50 kg weight category. As shown in Figure 1 athletes from −50 kg were mesomorphic ectomorphs while others showed endomorphic mesomorph somatotype. In addition, athletes from −78 kg and above 84 kg had an extreme endomorph–mesomorph rating, with the less expressed ectomorphic component. Contrary, female athletes in the lightest category were classified as endomorphic ectomorphs, athletes from next three categories were mesomorphic ectomorphs (−41, −44, and −48 kg), four weight category was endomorphic mesomorph (−52, −56, −60, and −65 kg) and two heaviest were classified as mesomorphic endomorphs. According to Drid et al,^[2]^ two separate homogeneous groups by weight division were noted in elite junior sambo athlete's booth gender. In regard to gender and weight category, male sambo athletes were heavier, taller and demonstrated lower body fat, higher circumferences and bone diameters, higher values of mesomorpic component and hand-grip strength than female athletes. Obtained results are in accordance with results noted in elite judo athletes.^[20–23]^ Such information might be useful for coaches, strength and conditioning professionals and physiotherapists.^[14]^ Sambo athletes of both genders in heavier weight categories had a stronger hand grip compared with lighter athletes. Anthropometrical characteristics are very important for sambo athletes, as the fight entails physical contact in which greater strength may provide an advantage. Hand-grip strength is very important for taking a grip and throwing especially in combat sport, such as sambo. Furthermore, strength hand-grip tests can provide relevant information in the sambo athletes’ evaluation process.

The present study had some limitations. First limitation is the lack of possibility to compare our results with another study related to cadet sambo athletes. Secondly, concerning hand-grip strength, there is no mention about the number of left-handed athletes by each weight category. Finally, our results are obtained from a sample of elite athletes which limits generalization of our results to elite cadet sambo athletes.

## 5 Conclusion

This study might help in profiling cadet sambo athletes taking into account gender, age and weight category. The anthropometric profile of sambo athletes changed according to their weight category. Mesomorphy was the most dominant somatotype component in male athletes, while female had three different types of somatotype component in relation to weight category. In conclusion, we found differences in hand-grip strength related to weight category. Our results showed that hand-grip strength was increased mainly with weight category in both genders. However, there was no mention concerning the number of left-handed athletes in each weight category. These differences found in hand-grip strength among weight categories are probably linked to differences in muscle mass between them. Future studies should focus on somatotype and handgrip strength values of international compared to national level cadet sambo athletes. Finally, these findings indicate that somatotype and hand-grip strength are considerable factors to achieve success in relation to weight category. The study also presents age-related normative data of cadet sambo athletes.

## Author contributions

**Conceptualization:** Tatjana Trivic, Sergey Eliseev, Sergey Tabakov, Vuk Raonic, Cristina Casals, Dzenan Jahic, Damjan Jaksic, Patrik Drid.

**Data curation:** Tatjana Trivic, Cristina Casals, Patrik Drid.

**Formal analysis:** Tatjana Trivic, Sergey Eliseev, Cristina Casals, Damjan Jaksic, Patrik Drid.

**Investigation:** Tatjana Trivic, Sergey Eliseev, Sergey Tabakov, Vuk Raonic, Cristina Casals, Dzenan Jahic, Damjan Jaksic, Patrik Drid.

**Methodology:** Tatjana Trivic, Sergey Eliseev, Sergey Tabakov, Vuk Raonic, Cristina Casals, Dzenan Jahic, Damjan Jaksic, Patrik Drid.

**Project administration:** Tatjana Trivic, Vuk Raonic, Dzenan Jahic.

**Resources:** Sergey Eliseev, Sergey Tabakov, Patrik Drid.

**Software:** Tatjana Trivic, Cristina Casals, Damjan Jaksic.

**Supervision:** Sergey Eliseev, Sergey Tabakov, Patrik Drid.

**Validation:** Tatjana Trivic, Sergey Eliseev, Vuk Raonic, Cristina Casals, Dzenan Jahic, Damjan Jaksic.

**Writing – original draft:** Tatjana Trivic.

**Writing – review & editing:** Sergey Eliseev, Sergey Tabakov, Vuk Raonic, Cristina Casals, Dzenan Jahic, Damjan Jaksic, Patrik Drid.

Patrik Drid orcid: 0000-0002-2075-6038.
